# Single Injection of High Volume of Autologous Pure PRP Provides a Significant Improvement in Knee Osteoarthritis: A Prospective Routine Care Study

**DOI:** 10.3390/ijms20061327

**Published:** 2019-03-15

**Authors:** Caroline Guillibert, Caroline Charpin, Marie Raffray, Annie Benmenni, Francois-Xavier Dehaut, Georges El Ghobeira, Roch Giorgi, Jeremy Magalon, Denis Arniaud

**Affiliations:** 1 Rheumatology Department, Hôpital Saint Joseph, 13008 Marseille, France; cguillibert@hopital-saint-joseph.fr (C.G.); ccharpin@hopital-saint-joseph.fr (C.C.); mraffray@hopital-saint-joseph.fr (M.R.); anbenmenni@hopital-saint-joseph.fr (A.B.); darniaud@hopital-saint-joseph.fr (D.A.); 2 Radiology Department, Hôpital Saint Joseph, 13008 Marseille, France; fxdehaut@hopital-saint-joseph.fr; 3 Physical Therapy Department, Hôpital Saint Joseph, 13008 Marseille, France; gelghobeira@hopital-saint-joseph.fr; 4 Aix Marseille Univ, APHM, INSERM, IRD, SESSTIM, Sciences Economiques et Sociales de la Santé & Traitement de l’Information Médicale, Hop Timone, BioSTIC, Biostatistique et Technologies de l’Information et de la Communication, 13005 Marseille, France; Roch.GIORGI@ap-hm.fr; 5 Cell Therapy Department, Hôpital de la Conception, AP-HM, INSERM CIC BT 1409, 13005 Marseille, France

**Keywords:** PRP, knee arthrosis, growth factors

## Abstract

Background: Evidence is growing regarding the ability of platelet-rich plasma (PRP) injections to enhance functional capacity and alleviate pain in knee osteoarthritis (OA). However, heterogeneity in common practice regarding PRP preparation and biological content makes the initiation of this activity in a hospital complex. The aim of this study was to document the efficacy of a single PRP injection to treat knee OA and validate a routine care procedure. Methods: Fifty-seven patients with symptomatic knee OA received a single injection of large volume of very pure PRP. They were assessed at baseline and after one, three and six months, by measuring Knee Injury and Osteoarthritis Score (KOOS), Observed Pain after a 50-foot walk test and Visual Analog Scale (VAS) assessments. Magnetic Resonance Imaging (MRI) analysis was performed at baseline and six months after the procedure. The objective was to recover 50% of responders three months after the procedure using OMERACT-OARSI criteria. Results: A single administration of high volume pure PRP provided significant clinical benefit for 84.2% of the responders, three months after the procedure. The KOOS total score significantly increased from 43.5 ± 14.3 to 66.4 ± 21.7 six months after the procedure (*p* < 0.001). Pain also significantly decreased from 37.5 ± 25.1 to 12.9 ± 20.9 (*p* < 0.001). No difference was observed on MRI parameters. Conclusion: A single injection of large volume of very pure PRP is associated with significant functional improvement and pain relief, allowing initiation of daily PRP injection within our hospital.

## 1. Introduction

Osteoarthritis (OA) of the knee is a progressive joint disease involving the intra-articular (IA) tibiofemoral and patellofemoral cartilage and all surrounding IA and periarticular structures [1]. In the United States, symptomatic OA affects more than 50 million adults, resulting in annual costs exceeding $ 100 billion due to medical expenses and lost wages [2]. Non pharmacological approaches such as exercise and lifestyle modifications are often associated with poor compliance [3] and pharmacological therapies (including analgesics, non-steroid anti-inflammatory drugs and corticosteroid injections) are not curative and induce side effects in the long term [4,5]. Hyaluronic acid (HA) injections were described as more effective in the long term compared to corticosteroids injection [6] with highly variable association with pain [7]. However, the American Academy of Orthopaedic Surgeons (AAOS) clinical practice guidelines provide evidence against HA viscosupplementation injections in patients with symptomatic knee OA [8]. This situation has led to the emergence of cell-based therapy options also called orthobiologics. Among them, platelet-rich plasma (PRP) is defined as an autologous plasma suspension of platelets, characterized by a platelet concentration higher than in physiological blood [9] and able to release growth factors (GFs) involved in reparative and regenerative processes. PRP has been the subject of increased clinical interest in the orthopaedic field and a recent meta analysis indicates that, compared with HA and saline, an intra-articular PRP injection may have more benefits in pain relief and functional improvement in patients with symptomatic knee OA at one year post-injection [10], providing orthopaedic surgeons and rheumatologists a new validated non-invasive option to improve knee OA related symptoms [11]. However, there is significant variation in the production of autologous PRP, including heterogeneity in the harvesting method, the type of anticoagulant used and the production method. Substantial differences in the content of platelets concentrates produced by the various automated and manual protocols have been described [12,13] and their consequences on clinical results in PRP therapy is currently being investigated [14,15]. Thus, the initiation of PRP injection activity within a hospital necessitates the selection of dedicated material and the establishment of a precise patient care procedure which should take into account recent recommendations from the AAOS regarding minimum reporting standards for clinical studies evaluating PRP [16].

In this context, the aim of this study was to document the efficacy of a single PRP injection to treat knee OA in routine care procedure. We assumed that PRP would allow to recover 50% of responders three months after treatment, using OMERACT-OARSI criteria. We also analyzed the relationship between clinical results and PRP composition through a precise biological characterization of the injected PRP, including platelets, leukocytes and red blood cells (RBCs) counts.

## 2. Results

### 2.1. Characteristics of Patients

**Figure 1:**
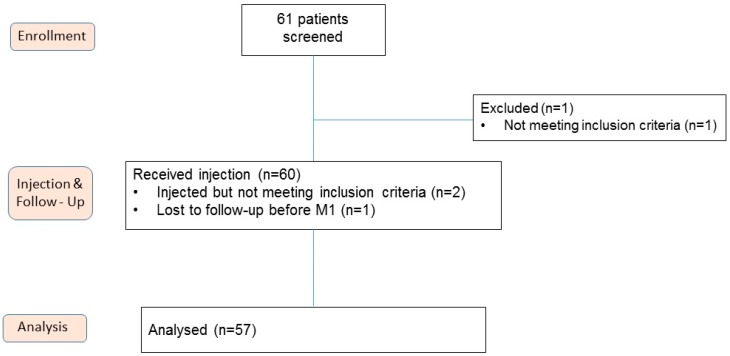
Flow diagram of the clinical trial. M1, month 1.

Out of 61 screened patients, 60 were injected and 57 were finally analyzed. One patient was originally included in the study population but finally excluded before the injection. Three patients were injected but could not be included in the analysis (one patient with lost follow up after one month and two patients with misunderstanding in inclusion criteria) (Figure 1). Patients were aged 63.3 ± 9.6 years old and presented grade 2 (40.3%) and grade 3 (59.7%) knee OA according to the Kellgren-Laurence scale (Table 1). 42 (73.7%) and 6 (10.5%) patients had previously had IA injection of HA or corticosteroids, respectively.

### 2.2. Biological Characteristics of PRP

Table 2 summarizes the biological characteristics of injected PRP. The final injected volume was 8.8 ± 1.1 mL. The mean recovery rate in platelets was 68.3 ± 16.5%. The mean increases in platelets and leukocytes compared with blood were 1.4 ± 0.4 and 0.1 ± 0.1, respectively. The percentage of platelets was 96.2 ± 2.5% with low contamination from RBCs (3.7 ± 2.4%) and leukocytes (0.1 ± 0.1%). The mean number of injected platelets was 2.5 ± 0.5 billion resulting in very pure PRP and a CCA rate according to DEPA (Dose, Efficiency, Purity, Activation) classification [17].

### 2.3. Effect of Single Autologous PRP Injection in Knee OA

**Figure 2:**
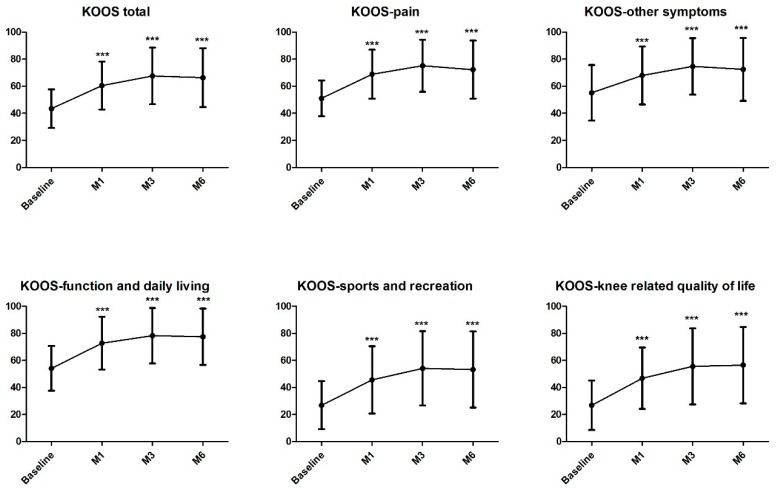
Evolution of KOOS total score and subscales. All follow-ups were statistically significant in comparison to baseline (***: *p* < 0.001; see Supplemental Table S1). KOOS, Knee Injury and Osteoarthritis Score. M1, month 1; M3, month 3; M6, month 6.

The main objective of the study was reached with 84.2% of OMERACT-OARSI responders at three months. This result was stable with 82.5% and 80.7% of responders one and six months after the injection, respectively. Single injection of PRP was effective in improving knee functional status with a significant increase in KOOS total score from 43.5 ± 14.3 to 66.4 ± 21.7, six months after the procedure (*p* < 0.001, Figure 2). Interestingly, this significant difference was observed already after one month after the treatment and was reflected on all KOOS subscores (Figure 2 and Supplemental Table S1). Assessment of pain through a 50-foot walk test also resulted in significant decrease of pain from the baseline (37.5 ± 25.1) as of one month after the injection (20.2 ± 23.3; *p* < 0.001, Supplemental Table S1) with a continuous effect until six months (12.9 ± 20.9; *p* < 0.001, Supplemental Table S1). Assessment of damages caused by the arthrosis were also reduced from 62.3 ± 19.6 at baseline to 42.1 ± 42.5 at six months with significant reduction at all follow-up (*p* < 0.001; Supplemental Table S1). Physical component score from the SF-36 significantly improved at all follow-up (*p* < 0.001, Supplemental Table S1). However, mental component score from the SF-36 did not change, which is consistent with the lack of improvement relative to patients’ global health that was relatively good at the beginning of the study (72.1 ± 16.0) (Supplemental Table S1). 78.9% of the patients and 80.7% of the rheumatologists were either satisfied or very satisfied regarding the procedure six months after the injection. Evolution of MRI parameters are presented in Table 3 without significant change six months after injection. Regarding safety, four adverse events (AE) were reported in four patients. AE were shoulder pain in two cases, acute pulmonary edema and peripheral arterial obstructive disease. None were considered related to the study treatment.

## 3. Discussion

Single administration of high volume of autologous pure PRP provided significant clinical benefit to more than 80% of responders at three months according to OMERACT-OARSI definition, in patients presenting knee OA in stage 2 or 3 according Kellgren–Laurence scale. These results are consistent with meta-analysis results from Dai et al. indicating functional improvement and pain relief one year after PRP injection [8]. Our study targeted patients similar to Dai et al. meta-analysis i.e., over 50 years old, presenting stage 2 or 3 on the Kellgren–Laurence scale. However, the therapeutic schema was different as the 10 randomized clinical trial (RCT) selected in that meta-analysis included multiple PRP injections whereas one of the originality of our study was to perform a single large volume PRP injection. Interestingly, only 3/10 of these RCT injected 8 mL whereas other studies injected 3 to 5.5 mL. From our point of view, PRP preparation for OA knee injection should take into account the articular capacity of the knee in order to favor a better distribution of PRP throughout the joint. It was recently recommended that the volume for knee-specific injection should be at 9 mL [18]. Thus, the medical device used in this study was validated in order to reach this condition. It was also selected to provide a pure PRP with limited contamination in RBCs and leukocytes and a platelets dose of around 2.5 billion in accordance with the results of Louis et al. [19]. Indeed, this study demonstrates that a single injection of very pure PRP (mean platelets dose ± SD of 2.4 ± 0.8 billion and platelets purity of 91.4 ± 4.1%) offers a significant clinical improvement equivalent to a single HA injection. This RCT also identified that higher platelets doses were correlated to higher levels of Transforming Growth Factor-β1 and Platelets Derived Growth Factor-AB as well as with knee function impairment, hence supporting limiting the injected dose of platelets. In our study, comparison between biological characteristics of PRP injected between OMERACT-OARSI responders (*n* = 48) and non-responders (*n* = 9) patients was performed without noticeable difference. This could be explained by (i) the small number of non-responder patients, (ii) the high reproducibility of the PRP produced in this study and (iii) the absence of GF quantification. This also suggests that this kind of correlation should be performed in RCT to limit the impact of placebo effect in the interpretation of biological data. Despite the excellent results of our study on pain and knee function, no statistical difference was observed compared to baseline in the parameters assessed by MRI. These results are consistent with the study of Buendia-Lopez et al. [20] who also assessed the articular thickness in all knee articular compartments and reached a similar conclusion. However, Lisi et al. [21] used a different methodology where they compared the number of patients with at least one grade improvement using MRI, according the Shahriaree classification system [22], six months after PRP or HA injections. They describe a significant difference with 16/31 patients reaching this improvement in PRP group and 8/31 in HA group, suggesting that guidelines for MRI interpretation in the context of regenerative medicine are needed.

The major limitation of our study is the absence of a control group which is an important weakness knowing the important placebo effect on arthrosis care. Indeed, it is important to recall that placebo can relieve pain in OA patients (effect size of 0.51) or improve function and stiffness (effect size of 0.49 and 0.43), particularly when administered through injections [23]. The absence of a placebo group was a conscious decision as many patients are discouraged to participate to a RCT involving PRP whereas it is daily and routinely injected in several rheumatology and orthopedic departments. Also, an adequately resourced RCT would require a higher number of patients and entail higher costs. Finally, it would have been interesting to document efficacy and MRI parameters one year after the injection, and extend the characterization of PRP to GF contents.

To conclude, our study indicates that a single injection of a large volume of very pure PRP is associated with a responders’ rate of around 80%, up to six months after the injection, supporting the initiation of daily PRP injection in OA patients in Saint Joseph hospital. 

In light of the growing evidence of its benefit and taking into account the current complex and heterogeneous context, a feasibility study according to AAOS guidelines would be desirable or even mandatory, prior to initiating regular PRP injection in OA.

## 4. Materials and Methods

### 4.1. Patients

To obtain a homogeneous study population, the patient suitability for inclusion was assessed (after a first screening by a physician), according to the following inclusion criteria: Age between 20 and 80 years old, symptomatic knee osteoarthritis grade 2 or 3 in Kellgren–Lawrence scale and according to the criteria of the American College of Rheumatology, axial deformity of the lower limb equal to or lower than 5°, at least moderate pain and difficulty to walk on a plain surface, ability to perform rehabilitation exercises, articular pain > 6 months, Hemoglobin (Hb) >10 g/dL, negative pregnancy test and written informed consent. The exclusion criteria were: Axial deformity of the lower limb over 5°, knee instability, important knee injuries or knee surgery less than 52 weeks before inclusion, Body Mass Index >35, thrombocytopenia <150 G/L or >450 G/L, thrombopathy, Hb <10 g/dL, infectious disease or positive serology to HIV, HCV, HBV and syphilis, actual chronic treatment by oral corticosteroid (or last dose taken less than two weeks before), intra articular knee injection of corticosteroid less than eight weeks before inclusion, intra articular knee injection of hyaluronic acid less than 24 weeks before inclusion, NSAID or anti platelets treatment completed less than two weeks before inclusion, fever or recent disease, auto immune disease, inflammatory arthritis, immune deficit, pregnancy and patient under guardianship or involved in another clinical trial. 

### 4.2. Study Design and Intervention

This monocentric prospective routine care trial was performed in the rheumatology department of Saint Joseph Hospital between March 2016 and August 2018. Patients received a single injection of PRP. After the injection, patients returned home with instructions to restrict movement of the leg for at least 48 hours and to use paracetamol or ice on the injected area to relieve pain if necessary. Non-steroidal anti-inflammatory drugs were prohibited during seven days following the injection. During the follow-up, no treatment restriction was applied and subsequently a gradual resumption of normal sport or recreational activities was tolerated.

The protocol was approved by the ethics committee and national health regulatory authority (CPP Ile de France 1 authorization #14243ND, 15th November 2016). The study was carried out in accordance with the Declaration of Helsinki and the principles of Good Clinical Practice. All patients gave written informed consent before their participation. The study was registered at clinicaltrials.gov (NCT 03082430).

### 4.3. Autologous PRP Preparation Method

After a four-fold skin decontamination (antiseptic foaming solution, rinsing with sterile water, drying, and alcoholic dermal antiseptic), a nurse collected 18 mL of blood by venipunture using a 21-gauge needle filling one 20 mL syringe containing 2 mL of ACD-A (Fidia, Abano Terme, Itlay). The blood was transferred into the Hy-tissue 20 PRP device (Fidia, Abano Terme, Itlay) before centrifugation using the Omnigrafter 3.0 (Fidia, Abano Terme, Itlay) and PRP Large Volume Cycle (3200 rpm during 10 minutes). All plasma was recovered using a 10 mL syringe through the Push-out system. 300 µL of whole blood and each autologous PRP preparation were sampled to determine platelets, leukocytes and RBCs concentrations using automated haematology blood cell analyzers Beckman Coulter DxH 801 (Beckman Coulter, Miami, FL, USA) according to recent guidelines [24].

### 4.4. Injection

The intra articular knee injection was performed with a 21 Gauge needle after conventional skin aseptic decontamination and under echographic control (HITACHI Aloka F37 Hitachi Aloka Medical Systems, Tokyo, Japan). 

### 4.5. Evaluation Tools and Follow-Up

Patients were prospectively assessed at baseline and one, three, and six months after injection. Evaluation included the Knee Injury and Osteoarthritis Score (KOOS), Observed Pain on 50-foot walk test, Visual Analog Scale (VAS) assessments (0–100 mm scale) during the previous week regarding arthrosis activity, damages related to arthrosis and global health. Quality-of-life was evaluated using the 36-Item Short Form Health Survey (SF-36) which was divided into two summary measures: The Physical (PCS) and Mental (MCS) Component Summary scores. Magnetic Resonance Imaging (MRI) analysis was performed at baseline and six months after the procedure using 1.5T MAGNETOM Aera and Avanto^fit^ (Siemens Healthcare, Erlangen, Germany). The presence of edema and joint effusion was graded as follows: 0 (absence), 1 (low), 2 (moderate), 3 (severe). The highest articular thickness was evaluated on same sequences for six compartments: Internal and lateral femoro-tibial compartments on femur and tibial parts respectively and internal and lateral femoro-patellar compartments.

Patients and rheumatologists satisfaction were rated at six months on a 5-level scale: Not satisfied, Few Satisfied, Moderately Satisfied, Satisfied and Very satisfied. Adverse events were recorded. Primary endpoint measure was defined as the percentage of OMERACT-OARSI responders at three months. OMERACT-OARSI criteria define responders patients if they satisfy either of the following criteria: (i) High improvement (≥50% and absolute change ≥ 10) in pain domain (pain observed on 50-foot walk test) or function (KOOS function in daily living subscore) or (ii) improvement (≥20% and absolute change ≥ 10) in at least 2/3 of the following domains: Pain, function or patient’s global assessment (VAS assessment on arthrosis activity previous week).

### 4.6. Statistical Analysis

Data were analyzed according to the intention-to-treat principle. The continuous variables were described by their mean and standard deviation. The categorical variables were described by their size and percentage. Comparisons over time, between two or more time points, were done using statistical test for repeated measurements. For situations involving two time points, we used Chi-square test, or Fisher’s exact test if the expected count in any cell was <5 for categorical data and Wilcoxon’s for continuous data. Correlation between covariates were quantified and tested using Pearson coefficient correlation. Friedman’s test was used to consider trend over times. The tests were performed bilaterally and were considered statistically significant when *p*-values ≤ 0.05. To deal with multiple testing in trend analysis, we used Holm’s method to produce *p*-values adjusted. The statistical analysis was performed with R software (version 3.1.0).

## Figures and Tables

**Table 1:** Baseline characteristics of patients included (*n* = 57) in statistical analysis.

Sex, male:female, *n*	24:33
Age, year, mean ± SD	63.3 ± 9.6
BMI, kg/m^2^, mean ± SD	25.4 ± 3.9
Symptom duration, mo, mean ± SD	8.4 ± 9.9
Kellgren Laurence Grade of knee OA grade 2: grade 3, *n*	23:34
Previous corticosteroids injection: HA injection, *n*	6:42
Flexion, °, mean ± SD	124.1 ± 10.3
Extension, °, mean ± SD	15.0 ± 4.6
KOOS total score, mean ± SD	43.9 ± 14.6
KOOS other symptoms score, mean ± SD	56.0 ± 20.9
KOOS pain score, mean ± SD	51.3 ± 13.3
KOOS function in daily living score, mean ± SD	54.5 ± 17.1
KOOS sport and recreation score, mean ± SD	27.5 ± 19.0
KOOS quality of life, mean ± SD	30.3 ± 18.0
Observed Pain on 50-foot walk test (0–100), mean ± SD	38.6 ± 25.3
Previous week VAS arthrosis activity (0–100)	57.9 ± 18.4
Previous week VAS damages caused by arthrosis (0–100)	63.0 ± 19.3
Previous week VAS global health (0–100)	72.6 ± 16.2
SF-36 PCS (0–100)	37.1 ± 9.3
SF-36 MCS (0–100)	40.4 ± 8.9

SD, standard deviation; BMI, body mass index; mo, months; OA, osteoarthritis; HA, hyaluronic acid; KOOS, Knee Injury and Osteoarthritis Score; VAS, visual analog scale; SF-36, Short Form Health Survey; PCS, Physical Component Summary; MCS, Mental Component Summary.

**Table 2:** Biological characteristics of platelet-rich plasma (PRP) (*n* = 57).

	Mean ± Standard Deviation
**Blood**	
Volume of whole blood collected, mL	18.0 ± 0.0
Red Blood Cells concentration, T/L	4.22 ± 0.65
Platelets concentration, G/L	197 ± 37
Leukocytes concentration, G/L	5.71 ± 1.22
**PRP**	
Volume of PRP injected, mL	8.8 ± 1.1
Red Blood Cells concentration, T/L	0.01 ± 0.01
Platelets concentration, G/L	288 ± 95
Leukocytes concentration, G/L	0.22 ± 0.27
Quantity of injected Red Blood Cells, millions	92 ± 53
Quantity of injected Red Blood Cells (%)	3.7 ± 2.4
Quantity of injected Platelets, millions	2517 ± 812
Quantity of injected Platelets (%)	96.2 ± 2.5
Quantity of injected Leukocytes, millions	2 ± 2
Quantity of injected Leukocytes (%)	0.1 ± 0.1
Recovery rate in platelets (%)	68.3 ± 16.5
Increase factor in platelets	1.4 ± 0.4
Increase factor in leukocytes	0.1 ± 0.1
DEPA Classification	CDA

**Table 3:** MRI assessment pre-injection and six months post-injection of PRP (*n* = 49).

	Pre-Operative	6 Months Post Injection	*p*
**Presence of edema** **(number of patients)**			
Grade 0	23	26	*p* = 1
Grade 1	18	13	
Grade 2	6	10	
Grade 3	2	8	
**Presence of joint effusion (number of patients)**			*p* = 1
Grade 0	14	14	
Grade 1	17	16	
Grade 2	14	13	
Grade 3	4	6	
**Articular thickness (mm, mean ± SD)**			
IFT-F compartment	1.16 ± 0.72	1.14 ± 0.77	*p* = 0.72
IFT-T compartment	1.67 ± 0.85	1.64 ± 0.89	*p* = 0.82
LFT-F compartment	1.60 ± 0.60	1.62 ± 0.60	*p* = 0.75
LFT-T compartment	2.08 ± 0.91	2.14 ± 1.01	*p* = 0.26
IFP compartment	2.27 ± 0.75	2.33 ± 0.77	*p* = 0.22
LFP compartment	2.61 ± 1.03	2.68 ± 1.06	*p* = 0.22

KOOS, Knee Injury and Osteoarthritis Score; I, internal; L, Lateral; FT, femorotibial; FP, femoropatellar; F, femur; T, tibia.

## Supplementary Materials

Supplementary materials can be found at https://www.mdpi.com/1422-0067/20/6/1327/s1.
