# Supplementary material for: Single Injection of High Volume of Autologous Pure PRP Provides a Significant Improvement in Knee Osteoarthritis: A Prospective Routine Care Study
Source: Int J Mol Sci. 2019 Mar 15;20(6):1327. doi: 10.3390/ijms20061327 (PMC6472196; doi:10.3390/ijms20061327)
Supplement: Supplementary file 1 [file ijms-20-01327-s001.pdf]

Supplemental table 1: Statistical Analysis of Secondary Efficacy outcomes (n=53)

KOOS, Knee Injury and Osteoarthritis Score; SF-36, Short Form Health Survey; PCS Physical Component Summary; MCS, Mental Component Summary. Statistical Analysis were performed by comparison to baseline.

	Baseline	M1	M3	M6
KOOS total score, mean ± SD	43.5 ± 14.3	60.5 ± 17.7<br>*p<0.001*	67.6 ± 20.9<br>*p<0.001*	66.4 ± 21.7<br>*p<0.001*
KOOS other symptoms score, mean ± SD	55.2 ± 20.5	68.0 ± 21.4<br>*p<0.001*	74.7 ± 20.9<br>*p<0.001*	72.5 ± 23.3<br>*p<0.001*
KOOS pain score, mean ± SD	51.1 ± 13.2	68.9 ± 18.2<br>*p<0.001*	75.2 ± 19.2<br>*p<0.001*	72.3 ± 21.4<br>*p<0.001*
KOOS function in daily living score, mean ± SD	54.1 ± 16.6	72.7 ± 19.5<br>*p<0.001*	78.3 ± 20.5<br>*p<0.001*	77.5 ± 20.8<br>*p<0.001*
KOOS sport and recreation score, mean ± SD	26.9 ± 17.8	45.6 ± 24.8<br>*p<0.001*	54.1 ± 27.5<br>*p<0.001*	53.3 ± 28.1<br>*p<0.001*
KOOS quality of life, mean ± SD	30.0 ± 18.3	46.8 ± 22.7<br>*p<0.001*	55.6 ± 28.1<br>*p<0.001*	56.5 ± 28.2<br>*p<0.001*
Observed Pain on 50-foot walk test (0-100), mean ± SD	37.5 ± 25.1	20.2 ± 23.3<br>*p<0.001*	17.8 ± 26.0<br>*p<0.001*	12.9 ± 20.9<br>*p<0.001*
Previous week VAS arthrosis activity (0-100) , mean ± SD	56.9 ± 18.4	40.0 ± 24.8<br>*p<0.001*	34.8 ± 29.3<br>*p<0.001*	34.2 ± 28.7<br>*p<0.001*
Previous week VAS damages caused by arthrosis (0-100) , mean ± SD	62.3 ± 19.6	45.8 ± 23.3<br>*p<0.001*	38.5 ± 26.2<br>*p<0.001*	42.1 ± 24.5<br>*p<0.001*
Previous week VAS global health (0-100) , mean ± SD	72.1 ± 16.0	74.0 ± 17.5<br>p=1	75.6 ± 21.6<br>p=0.96	73.1 ± 24.4<br>p=1
SF-36 PCS (0-100) , mean ± SD	37.4 ± 9.3	43.7 ± 10.8<br>*p<0.001*	46.8 ± 11.8<br>*p<0.001*	46.1 ± 12.8<br>*p<0.001*
SF-36 MCS (0-100) , mean ± SD	40.1 ± 8.9	41.3 ± 7.4<br>p=1	41.3 ± 7.8<br>p=1	42.5 ± 7.4<br>p=0.72
